# Combined effects of glutathione S-transferase M1 and T1 polymorphisms on risk of lung cancer: evidence from a meta-analysis

**DOI:** 10.18632/oncotarget.15943

**Published:** 2017-03-06

**Authors:** Ying Gao, Fei Gao, Ting-Ting Hu, Gang Li, Yan-Xia Sui

**Affiliations:** ^1^ Department of Radiotherapy Oncology, First Affiliated Hospital of Medical College of Xi’an, Jiao Tong University, Xi’an, Shanxi, China; ^2^ Department of Neurology, First Affiliated Hospital of Xi’an Medical University, Xi’an, Shanxi, China; ^3^ Department of Medical, First Affiliated Hospital of Medical College of Xi’an, Jiao Tong University, Xi’an, Shanxi, China; ^4^ Second Department of Thoracic Surgery, First Affiliated Hospital of Medical College of Xi’an, Jiao Tong University, Xi’an, Shanxi, China; ^5^ Department of Pathology, First Affiliated Hospital of Medical College of Xi’an, Jiao Tong University, Xi’an, Shanxi, China

**Keywords:** GSTM1, GSTT1, polymorphism, lung cancer, meta-analysis

## Abstract

Many studies have reported an association between the glutathione S-transferase M1 null and T1 null polymorphisms and lung cancer risk. However, the combined effects of *GSTM1* null and *GSTT1* null polymorphisms have not been reported previously. We, therefore, performed a meta-analysis to investigate the combined effects. 40 publications with 44 case–control studies were selected in the meta-analysis, including 13,706 cases and 13,093 controls. Significant association was observed between the combined effects of *GSTM1* and *GSTT1* polymorphisms and lung cancer risk when all the eligible studies were pooled into the meta-analysis. When we performed subgroup analysis, significantly increased lung cancer risk was observed in Caucasians (− − vs. + +: OR = 1.23, 95% CI: 1.07 to 1.41), Asians (− − vs.− +: OR = 1.24, 95% CI: 1.10 to 1.41; recessive model: OR = 1.45, 95% CI: 1.19 to 1.77; dominant model: OR = 1.53, 95% CI: 1.24 to 1.90), Indians (− − vs. + +: OR = 2.53, 95% CI: 1.61 to 3.98; recessive model: OR = 1.69, 95% CI: 1.07 to 2.67; dominant model: OR = 2.11, 95% CI: 1.36 to 3.28), hospital-based studies, and population-based studies. In summary, this meta-analysis indicates that the combined effects of the *GSTM1* and *GSTT1* polymorphisms are associated with increased lung cancer risk in Asians, Caucasians, and Indians.

## INTRODUCTION

Lung cancer has become the most common cancer and the leading cause of cancer death in the world [1]. There were about 219,440 lung cancer cases and 159,390 deaths expected in the United States in 2009 [2]. In recent years, the incidence of lung cancer in Asia increased rapidly [3]. Tobacco smoking is clearly the strongest risk factor for lung cancer [4]. However, not all smokers develop lung cancer, which indicates that other causes, including genetic susceptibility, may contribute to development of lung cancer [5–6].

Possible lung cancer susceptibility genes have been sought among tumor suppressor genes, DNA repair genes, and genes encoding phase I and phase II enzymes [7]. Glutathione S-transferases (GSTs) comprise a multi-gene family encoding enzymes, is phase II transformation enzymes involved in the detoxification of hazardous agents [8]. Acting nonenzymically, GSTs can modulate signalling pathways of cell proliferation, cell differentiation and apoptosis [9]. GSTs can be classified into at least four genetically distinct groups including Glutathione S-transferase M1(GSTM1) and Glutathione S-transferase T1(GSTT1) [10]. *GSTM1* and *GSTT1* are two of the most important GST variants. GSTM1 located on chromosome 1p13.3 with 10 exons and *GSTT1* is mapped at chromosome 22q11.23 and contains six exons. Individuals with the homozygous deletion of the *GSTM1* and *GSTT1* locus (*GSTM1* null and *GSTT1* null) have no enzymatic functional activity [11].

To date, some previous meta-analyses have only studied the association between *GSTM1* and/or *GSTT1* alone gene polymorphisms and risk of lung cancer [12–14, 63, 64]. However, the combined effects of *GSTM1* and *GSTT1* polymorphisms have not been reported previously. In addition, a number of molecular epidemiological studies have been performed to evaluate the association between the combined effects of glutathione S-transferase M1 and T1 polymorphisms and lung cancer risk [15–56]. However, the results were inconsistent or even contradictory. Therefore, we performed a meta-analysis to investigate the combined effects of *GSTM1* and *GSTT1* and lung cancer risk.

## RESULTS

### Literature search and meta-analysis databases

**Figure 1:**
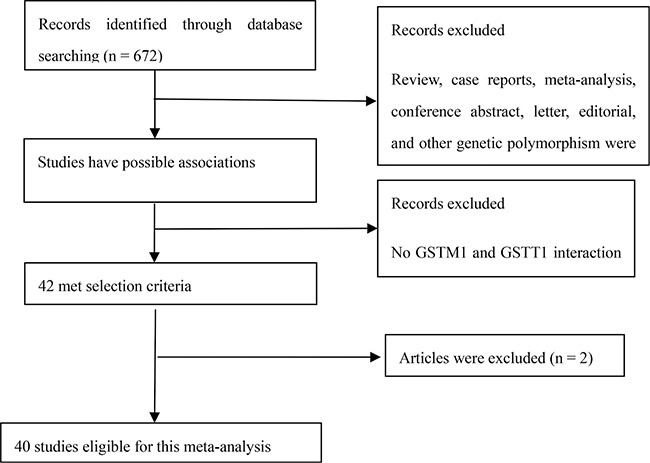
Flow diagram of selection process to identify eligible studies in the meta-analysis

Relevant publications were retrieved and preliminarily screened. As shown in Figure 1, 672 potentially relevant publications were identified. Of these, 549 publications were excluded after reading the title or abstract because of obvious irrelevance with our study aim (this included review, case reports, meta-analysis, conference abstract, letter, editorial, and other genetic polymorphism). In addition, of these published articles, 2 articles [49, 51] were excluded because of their sample size overlapped with another 2 articles [41, 27]. As summarized in Supplementary Table 1, 40 publications with 44 case–control studies were selected for the meta-analysis, including 13,706 cases and 13,093 controls. Among these publications, 21 were Caucasians, 14 were Asians, 3 were Indians, 3 were Africans, and 3 were mixed populations. In addition, 7 were Chinese articles and 37 were English articles. Last, 31 were hospital-based studies and 13 were population-based studies.

### Quantitative synthesis

**Figure 2:**
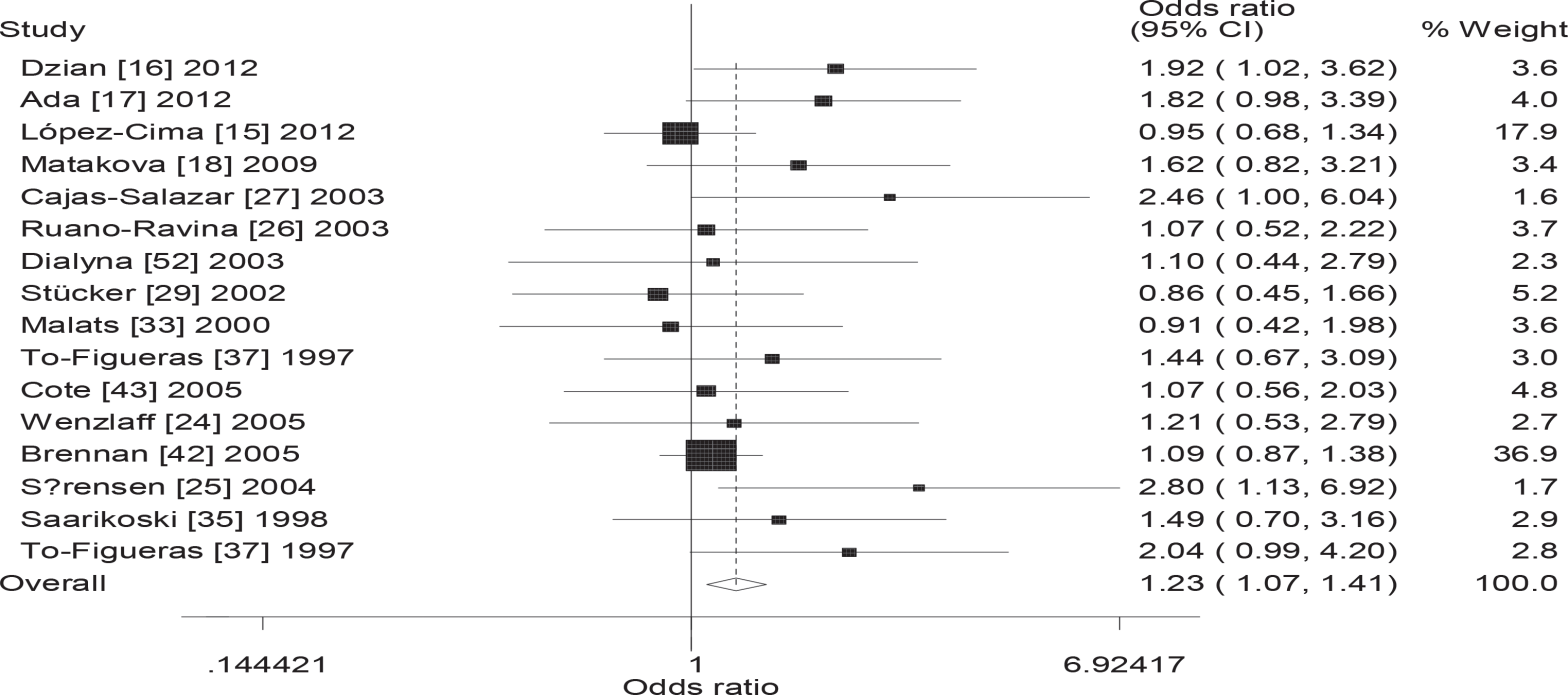
Forest plot of the the combined effects of GSTM1 and GSTT1 polymorphisms and lung cancer risk in Caucasians (− − vs. + +)

**Figure 3:**
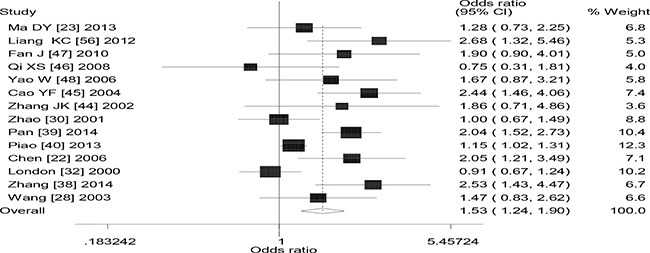
Forest plot of the the combined effects of GSTM1 and GSTT1 polymorphisms and lung cancer risk in Asians (dominant model)

Table 1 lists the main results of the meta-analysis of the association between the combined effects of *GSTM1* and *GSTT1* polymorphism and lung cancer risk. Significant association was observed between the combined effects of *GSTM1* and *GSTT1* polymorphism and lung cancer risk (− − vs. + +: OR = 1.58, 95% CI: 1.34 to 1.87, *P* < 0.001; − − vs. + −: OR = 1.26, 95% CI: 1.13 to 1.42, *P* < 0.001; − − vs.− +: OR = 1.26, 95% CI: 1.08 to 1.48, *P* = 0.004; recessive model: OR = 1.27, 95% CI: 1.13 to 1.42, *P* < 0.001; dominant model: OR = 1.33, 95% CI: 1.19 to 1.48, *P* < 0.001) when all the eligible studies were pooled into the meta-analysis. Significant between-study heterogeneity was detected in overall analysis. Hence, then we performed subgroup analysis by ethnicity, significantly increased lung cancer risk was observed in Caucasians (− − vs. + +: OR = 1.23, 95% CI: 1.07 to 1.41, *P* = 0.003, Figure 2), Asians (− − vs.− +: OR = 1.24, 95% CI: 1.10 to 1.41, *P* = 0.001; recessive model: OR = 1.45, 95% CI: 1.19 to 1.77, *P* < 0.001; dominant model: OR = 1.53, 95% CI: 1.24 to 1.90, *P* < 0.001, Figure 3), Indians (− − vs. + +: OR = 2.53, 95% CI: 1.61 to 3.98, *P* < 0.001; recessive model: OR = 1.69, 95% CI: 1.07 to 2.67, *P* = 0.025; dominant model: OR = 2.11, 95% CI: 1.36 to 3.28, *P* = 0.001). In addition, we also performed subgroup analysis by source of controls, significantly increased lung cancer risk was observed in hospital-based studies (− − vs. + +: OR = 1.58, 95% CI: 1.29 to 1.94, *P* < 0.001; recessive model: OR = 1.30, 95% CI: 1.14 to 1.48, *P* < 0.001; dominant model: OR = 1.36, 95% CI: 1.18 to 1.56, *P* < 0.001) and population-based studies (− − vs. + +: OR = 1.58, 95% CI: 1.16 to 2.14, *P* < 0.001; − − vs.− +: OR = 1.48, 95% CI: 1.12 to 1.94, *P* = 0.006; recessive model: OR = 1.24, 95% CI: 1.04 to 1.49, *P* = 0.016; dominant model: OR = 1.29, 95% CI: 1.07 to 1.55, *P* = 0.006).

**Table 1:** Combined genotype analysis of GSTM1 and GSTT1on risk of lung cancer

Variables	Studies	Cases/control	Test of association				Test of heterogeneity			Model
OR	95% CI	Z	P	Chi-squared	*P*_h_	*I*^2^ (%)
− − vs. + +										
Overall	34	5,886/5,224	1.58	1.34–1.87	5.34	< 0.001	78.12	< 0.001	57.8	Random effect model
Subgroup analyses by ethnicity										
Caucasian	16	2,608/2,893	1.23	1.07–1.41	2.95	0.003	17.04	0.317	12.0	Fixed effect model
Asian	11	2,707/1,674	–	–	–	–	42.92	< 0.001	76.7	–
Indian	3	348/391	2.53	1.61–3.98	4.02	< 0.001	1.50	0.473	0.0	Fixed effect model
Subgroup analyses by study design										
HB	23	1,971/2,242	1.58	1.29–1.94	4.38	< 0.001	38.11	0.018	42.3	Random effect model
PB	11	3,915/2,982	1.58	1.16–2.14	2.93	0.003	38.98	< 0.001	74.3	Random effect model
− − vs. + −										
Overall	23	3,309/2,063	1.26	1.13–1.42	4.02	< 0.001	23.08	0.397	4.7	Fixed effect model
− − vs. − +										
Overall	23	4,447/3,198	1.26	1.08–1.48	2.91	0.004	32.11	0.076	31.5	Random effect model
Subgroup analyses by ethnicity										
Caucasian	10	1,263/1,400	1.15	0.86–1.56	0.94	0.346	17.52	0.041	48.6	Random effect model
Asian	10	2,948/1,592	1.24	1.10–1.41	3.42	0.001	13.48	0.142	33.2	Fixed effect model
Subgroup analyses by study design										
HB	17	1,514/1,569	1.16	0.98–1.37	1.76	0.078	21.78	0.150	26.5	Fixed effect model
PB	6	2,933/1,629	1.48	1.12–1.94	2.77	0.006	9.91	0.078	49.5	Random effect model
− − vs. (+ −) + (− +)										
Overall	34	8,177/6,586	1.27	1.13–1.42	4.19	< 0.001	45.99	0.066	28.2	Random effect model
Subgroup analyses by ethnicity										
Caucasian	16	3,417/3,558	1.09	0.97–1.23	1.40	0.163	20.04	0.170	25.1	Fixed effect model
Asian	11	4,159/2,403	1.45	1.19–1.77	3.72	< 0.001	16.60	0.084	39.8	Random effect model
Indian	3	348/273	1.69	1.07–2.67	2.24	0.025	2.04	0.360	2.0	Fixed effect model
Subgroup analyses by study design										
HB	23	2,601/2,714	1.30	1.14–1.48	3.92	< 0.001	24.48	0.323	10.1	Fixed effect model
PB	11	5,576/3,867	1.24	1.04–1.49	2.41	0.016	19.85	0.031	49.6	Random effect model
− − vs. (+ −) + (− +) + (+ +)										
Overall	44	13,706/13,093	1.33	1.19–1.48	5.11	< 0.001	79.41	0.001	45.9	Random effect model
Subgroup analyses by ethnicity										
Caucasian	21	6,771/7,545	1.10	0.99–1.22	1.86	0.063	22.73	0.302	12.0	Fixed effect model
Asian	14	5,766/4,337	1.53	1.24–1.90	3.95	< 0.001	40.79	< 0.001	68.1	Random effect model
Indian	3	632/632	2.11	1.36–3.28	3.34	0.001	2.02	0.363	1.2	Fixed effect model
African	3	219/278	1.34	0.86–2.10	1.28	0.201	0.55	0.758	0.0	Fixed effect model
Subgroup analyses by study design										
HB	31	5,694/6,359	1.36	1.18–1.56	4.29	< 0.001	46.4	0.028	35.3	Random effect model
PB	13	8,012/6,734	1.29	1.07–1.55	2.72	0.006	31.87	0.001	62.3	Random effect model

+ − Refers to *GSTM1* Present *GSTT1* Null; − + refers to *GSTM1* Null *GSTT1* Present; − − refers to *GSTM1* Null *GSTT1* Null; + + refers to *GSTM1* Present *GSTM1* Present; HB hospital-based studies; PB population-based studies

### Heterogeneity and sensitivity analysis

Significant heterogeneity was observed in the meta-analysis, as shown in Table 1. Hence, we used meta-regression analysis to assess the source of heterogeneity by ethnicity, source of controls, and language. The results indicated that ethnicity, source of control, and language did not contribute to substantial heterogeneity in the meta-analysis. *I^2^ >* 75.0% was observed in Asians (− − vs.+ +), however, when the study of Cao YF et al. [45] was excluded, heterogeneity values dropped in Asians (− − vs. + +: *I^2^* = 72.5%). In addition, exclusion of the study of Cao YF et al. [45] resulted in a statistically significant association between the combined effects of *GSTM1* and *GSTT1* polymorphisms and lung cancer risk in Asians (− − vs. + +: OR = 1.91, 95% CI: 1.34 to 2.74, *P* < 0.001).

### Publication bias

**Figure 4:**
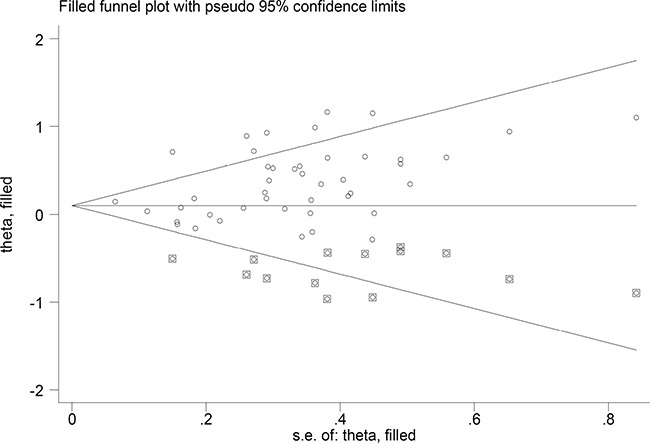
The Duval and Tweedie nonparametric “trim and fill” method's funnel plot of the meta-analysis (dominant model)

Using Egger's test, significant publication bias was detected in the meta-analysis (dominant model: *P* = 0.007). We used the Duval and Tweedie nonparametric ‘‘trim and fill’’ method to adjust for publication bias. Figure 4 lists the Duval and Tweedie nonparametric “trim and fill’’ methods funnel plot. The pooled results did not change between the combined effects of GSM1 and T1 polymorphisms and lung cancer risk in dominant model.

## DISCUSSION

Lung cancer, accounting for 13 % (1.6 million) of all cancers and 18 % (1.4 million) of all cancer deaths in 2008, has become the most common cancer in the world [1]. Gene polymorphism factor has been reported to be an important factor which increases the susceptibility of lung cancer. Some genetic polymorphisms alters the enzyme activity and leads to individual differences in the susceptibility to cancer [67]. *GSTM1* and *GSTT1* null genotype lost enzymatic functional activity, therefore, may increase the risk of lung cancer. To date, a series of previous meta-analysis have only focused on the association between single *GSTM1* or *GSTT1* gene polymorphism and lung cancer risk [12–14, 63, 64]. However, the combined effect of *GSTM1* and *GSTT1* has not been reported previously. In addition, many studies have been performed to evaluate the association between the combined effects of glutathione S-transferase M1 and T1 polymorphisms and lung cancer risk. However, the results were inconsistent or even contradictory. Therefore, we performed a meta-analysis to investigate the combined effects of *GSTM1* and *GSTT1* polymorphisms and lung cancer risk.

Significant association was found between the combined effects of *GSTM1* and *GSTT1* polymorphisms and lung cancer risk when all the eligible studies were pooled into the meta-analysis. When we performed subgroup analysis, significant increased lung cancer risk was observed in Caucasians, Asians, and Indians. It is possible that the effect sizes of genetic factors predisposing to human diseases are different in various ethnic populations [66]. Dzian et al. [16], Matakova et al. [18], and Cajas-Salazar et al. [27] suggested that the combined effects of GSTM1 and GSTT1 polymorphisms increased lung cancer risk in Caucasians. Chen et al. [22], Liang KC et al. [56], Cao YF et al. [45], Pan et al. [39], Piao et al. [40], and Zhang et al. [38] suggested that the *GSTM1* and *GSTT1* polymorphisms in combination were associated with increased risk factors of lung cancer in Asians. Sharma et al. [31] and Sreeja et al. [41] found that the combined effects of *GSTM1* and *GSTT1* polymorphisms increased lung cancer risk in Indians. The results of the meta-analysis supported the positive association. However, at any case, the association between the combined effects of *GSTM1* and *GSTT1* polymorphisms and lung cancer risk in Indians essentially remains an open field, as the number of studies (*n* = 3) is considerably smaller than that needed for the achievement of robust conclusions [65]. When we performed subgroup analysis by source of controls, significantly increased lung cancer risk was also observed in hospital-based studies and population-based studies. The hospital-based studies have some biases because such controls might be a sample of ill-defined reference population. Hence, using a proper population-based study was very important.

In this meta-analysis, several limitations should be acknowledged. First, due to the limited participant data provided by individual studies, we could not examine the interactions among gene-environment. Second, only published articles were included in this study, which may cause publication bias. Third, the controls were not uniformly defined. However, the current meta-analysis has also particular strengths. First, our meta-analysis explores and analyzes the sources of heterogeneity. Second, This is the first meta-analysis to explore the combined *GSTM1* and *GSTT1* effects and lung cancer risk. Third, a meta-analysis is statistically more powerful than any single study. Fourth, the quality of eligible studies met our inclusion criterion in the meta-analysis.

In summary, this meta-analysis indicates that the combined effects of *GSTM1* and *GSTT1* polymorphisms is associated with increased lung cancer risk in Asians, Caucasians, and Indians. As we only explored the association between two combined genes and lung cancer risk, there is still leaving much to be studied in depth. To further evaluate the effects of gene–gene interaction on lung cancer risk, a population-based study on large size of samples is required.

## MATERIALS AND METHODS

### Identification and eligibility of relevant studies

We searched the electronic databases including PubMed, Embase, Web of Science, and two Chinese databases China National Knowledge Infrastructure (CNKI) and Wanfang Data (the last search update was August 8, 2016) with The following key words (“Glutathione S-transferase M1 or “*GSTM1*”, “Glutathione S-transferase T1 or “*GSTT1*”, “variant” or “variation” or “polymorphism”, and “lung”. The search was performed without any restrictions on language. In addition to the electronic database search, we reviewed manually the reference lists of retrieved articles to identify additional articles.

### Inclusion criteria and data extraction

The included studies met the following criteria: (1) patients had a diagnosis of lung cancer; (2) evaluated combination effects of the *GSTM1* and *GSTT1* polymorphisms and lung cancer risk; (3) used case–control or cohort studies; (4) provided sufficient data of combined genotypes for the *GSTM1* and *GSTT1* polymorphisms in lung cancer patients and controls; (5) reviews, conference abstracts, letters, and editorials were excluded; (5) duplications of previously published data were excluded (if more than one article was published using the same case data, only the article with the largest sample size was selected). The following data were collected from each study: (1) first author's name; (2) year of publication; (3) country of origin; (4) ethnicity; (5) source of controls (population-based controls and hospital-based controls); (6) language; (7) numbers of cases and controls in the combined genotypes for the *GSTM1* and *GSTT1* polymorphisms whenever possible. Ethnicity was categorized as ‘‘Caucasian’’, ‘‘Asian’’, and “African” (including African Americans). We also considered the samples of studies from India and Pakistan as of “Indian”. Last, When one study did not state which ethnic groups was included, or if it was impossible to separate participants according to phenotype, the sample was termed as ‘‘mixed population’’.

### Statistical analysis

The association between the combined effects of *GSTM1* and *GSTT1* polymorphisms and lung cancer risk was calculated by the crude odds ratios (ORs) as the combined study effect size and summary statistics with 95 % confidence intervals (CI) in a dominant model (− − vs. (+ −) + (− +) + (+ +)), a recessive model (− − vs. (+ −) + (− +)), and a co-dominant model (− − vs. + +, − − vs. + −, and − − vs.− +). − − represented *GSTM1* null *GSTT1* null genotype. + − represented *GSTM1* present *GSTT1* null genotype. − + represented *GSTM1* null *GSTT1* present genotype. + + represented *GSTM1* present *GSTT1* present genotype. The significance of the pooled OR was determined by the *Z*-test, and *P* < 0.05 was considered as statistically significant. Statistical heterogeneity among studies was assessed by the chi-square-based *Q* test [57] and *I*^2^ value [58]. In heterogeneity tests, when *P* < 0.1, a random-effects model was used [59], otherwise a fixed-effects model was performed [60]. Meanwhile, if *I*^2^ ≥ 50%, 50% > *I*^2^ ≥ 25% or 0 < *I*^2^ < 25%, we identified the studies as high, middle or low heterogeneity, respectively. Publication bias was analyzed by Egger's linear regression test [61]. If publication bias existed, the Duval and Tweedie nonparametric “trim and fill” method was used to adjust for it [62]. Sensitivity analysis was performed by excluding one study at a time to evaluate the stability of the results. A meta-regression analysis was carried out to identify the major sources of between-studies variation in the results, using the log of the ORs from each study as dependent variables, and ethnicity, language and source of controls as the possible sources of heterogeneity. All statistical tests were performed by STATA version 10.0 (STATA Corporation, College Station, TX).

## SUPPLEMENTARY MATERIALS TABLE





## Abbreviations

GSTM1: glutathione S-transferase M1
OR: odds ratio
95% CI: 95% confidence interval
HB: hospital-based controls
PB: publication-based controls
