# Supplementary material for: Combined effects of glutathione S-transferase M1 and T1 polymorphisms on risk of lung cancer: evidence from a meta-analysis
Source: Oncotarget. 2017 Mar 6;8(17):28135–43. doi: 10.18632/oncotarget.15943 (PMC5438637; doi:10.18632/oncotarget.15943)
Supplement: Supplementary file 1 [file oncotarget-08-28135-s001.pdf]

Combined effects of glutathione S-transferase M1 and T1 polymorphisms on risk of lung cancer: Evidence from a meta-analysis

Supplementary Materials

Supplementary Table 1: The data between combined genes of *GSTM1* and *GSTT1* polymorphism and lung cancer risk. see Supplementary_Table_1
